# Prevalence and Molecular Epidemiology of Transmitted Drug Resistance and Genetic Transmission Networks Among Newly Diagnosed People Living With HIV/AIDS in a Minority Area, China

**DOI:** 10.3389/fpubh.2021.731280

**Published:** 2021-10-11

**Authors:** Dan Yuan, Bin Yu, Yiping Li, Zixin Wang, Meijing Liu, Li Ye, Yuling Huang, Ling Su, Yan Zhang, Laze Api, Maogang Chen, Chang Zhou, Li Liu, Linglin Zhang, Shu Liang, Peng Jia, Shujuan Yang

**Affiliations:** ^1^Center for AIDS/STD Control and Prevention, Sichuan Center for Disease Control and Prevention, Chengdu, China; ^2^West China Second University Hospital of Sichuan University and Key Laboratory of Birth Defects and Related Diseases of Women and Children (Sichuan University), Ministry of Education, Chengdu, China; ^3^Centre for Health Behaviours Research, The Jockey Club School of Public Health and Primary Care, The Chinese University of Hong Kong, Hong Kong, SAR China; ^4^West China School of Public Health and West China Fourth Hospital, Sichuan University, Chengdu, China; ^5^Butuo Center for Disease Control and Prevention, Liangshan, China; ^6^Liangshan Center for Disease Control and Prevention, Xichang, China; ^7^School of Resources and Environmental Science, Wuhan University, Wuhan, China; ^8^International Institute of Spatial Lifecourse Epidemiology (ISLE), Wuhan University, Wuhan, China

**Keywords:** antiretroviral therapy, HIV, mutations, transmitted drug resistance, molecular epidemiology

## Abstract

**Introduction:** Transmitted drug resistance (TDR) can compromise antiretroviral therapy (ART) efficacy. We aimed to understand the molecular epidemiology of TDR and its genetic transmission networks among newly diagnosed people living with HIV/AIDS (PLWH).

**Methods:** A total of 1,318 newly diagnosed PLWH, identified in all population-based HIV screening in an HIV-affected county of a minority area of China (i.e., Butuo county), were enrolled between January 1, 2018, and November 31, 2018. HIV-1 *pol* gene sequences were used for phylogenetic and genotypic drug resistance analyses. The genetic transmission networks were identified.

**Results:** The prevalence of TDR among newly diagnosed PLWH was 8.12% (107/1,318). Patients in the stage of AIDS (adjusted odds ratio, OR: 2.32) and who had a history of sharing a needle ≥5 times (adjusted OR: 3.89) were more likely to have an increased risk of TDR. The prevalence of TDR for non-nucleoside reverse transcriptase inhibitors (NNRTIs) is higher than that of other inhibitors, with a relatively high prevalence of three mutations [V179D/E/DE (4.93%), K103N/KN (3.11%), and E138A/G (1.52%)]. A total of 577 (43.78%) *pol* sequences were involved in the genetic transmission network, with 171 clusters ranging in size from 2 to 91 *pol* sequences; 37.38% (40/107) of individuals carrying TDR were involved in the network, and individuals with the same TDR-associated mutations were usually cross-linked.

**Conclusions:** Our data suggest a relatively high level of TDR and many transmission clusters among the newly diagnosed PLWH. Targeted intervention, early identification, and monitoring of resistance are warranted to reduce the TDR and prevent HIV-1 transmission in areas with a high rate of HIV-1.

## Introduction

The human immunodeficiency virus affected more than 37 million individuals worldwide, and there were approximately 0.85 million people living with HIV/AIDS (PLWH) in China as of 2018. The number of PLWH in Sichuan has been increasing dramatically in recent years, and a total of 161,456 PLWH cases were reported in 2018. Liangshan Yi Autonomous Prefecture (Liangshan), the largest traditional settlement of the Yi minority people in China, has the highest prevalence of HIV. Butuo county in Liangshan is one of the areas with the most serious HIV epidemic in China (the prevalence of HIV was over 1% in recent years) (1–3). Effective interventions are needed to reduce HIV transmissions in this area.

Antiretroviral therapy (ART) is effective in suppressing HIV, preventing the disease progression, and secondary HIV transmission (4, 5). However, antiretroviral resistance remains to be one of the barriers to sustaining HIV suppression during ART (6, 7). According to the origin of drug-resistant virus strains *in vivo*, resistance can be divided into transmitted drug resistance (TDR) and acquired drug resistance (ADR). ADR is defined as drug resistance that develops when HIV mutations emerge due to viral replication in individuals receiving antiretroviral drugs (8). TDR occurs when previously uninfected individuals are infected with drug-resistant HIV strains, and TDR is detected in PLWH with no history of antiretroviral drug exposure (8). Severe TDR is likely to occur in non-recently contaminated patients (9, 10), and the policy of treating all PLWH decreases the risk of transmission of resistant strains. The diversity of HIV-1 subtypes and recombinants is thought to contribute to the TDR levels (11). TDR may not only increase the risk of virologic failure on ART but also lead to the spread of drug-resistant strains, bringing a challenge in eliminating HIV. Therefore, TDR should be an intensive focus in HIV surveillance to prevent the spread of drug-resistant strains.

Genetic transmission networks are mainly focused on similar viruses with similarities and connections. Genetic distance-based methods describe a putative transmission link to any pair of viral sequences that are within a predetermined genetic distance threshold (12–14). The genetic relatedness of HIV-1 could partly reflect the unobserved relationship between PLWH in combination with an epidemiologic investigation, and the spread of TDR among them, based on which the potential genetic transmission networks can be referred (15, 16). Analysis of the genetic transmission networks among newly diagnosed PLWH is useful to understand TDR and provide information for the first-line ART strategy (15, 16). However, there is a dearth of studies investigating molecular epidemiology of TDR in China, and no study was conducted among PLWH in Liangshan (the prevalence of HIV in five counties was over 1%).

To fill the knowledge gap, we analyzed the molecular epidemiology of TDR and genetic transmission networks and evaluated the TDR-associated mutations in genetic transmission networks among newly diagnosed PLWH identified by all population-based HIV screening methods in Liangshan. The results would be useful for understanding TDR and improving HIV surveillance and public health intervention activities especially in areas seriously affected by HIV.

## Methods

### Study Participants

All subjects voluntarily participated in our study and signed informed consent forms before enrollment according to the Helsinki Declaration of 1964. For participants of age <16 years, we have obtained their informed consent from parents/guardians. The study protocol was approved by the Ethics Committee of Sichuan Center for Disease Prevention and Control. All methods were carried out in accordance with relevant guidelines and regulations.

A cross-sectional design was performed in this study. Butuo is one of the areas with the most serious HIV epidemic in China, and it is located in the middle east of Liangshan, Sichuan Province (Supplementary Figure 1). According to the implementation of the Health and Medical Assistance Program for Poverty Alleviation in Liangshan, residents in Butuo county were asked to receive HIV screening and physical examination (17). Since November 2017, the all-population HIV screening plan has been carried out in Butuo county. By the end of 2018, the total population reached 174,934 in Butuo county. After excluding PLWH identified before January 1, 2018, and individuals who could not be reached or refused to participate, a total of 160,956 individuals agreed to participate in the screening for HIV. A total of 2,259 newly diagnosed PLWH were identified between January 1, 2018, and November 31, 2018. The newly diagnosed PLWH were considered as study participants. After excluding those with a lack of or insufficient blood samples for sequencing or incomplete personal information, 1,595 newly diagnosed PLWH were enrolled in our study (Supplementary Figure 2).

A total of 1,595 newly diagnosed PLWH were enrolled according to the following inclusion criteria: (1) diagnosed with HIV/AIDS during the period from January 1, 2018, to November 31, 2018; (2) had no evidence of receiving ART before enrollment; (3) signed the informed consent for TDR testing; (4) the plasma samples were successfully collected to perform the sequencing.

### Data Collection

A questionnaire was used to privately ask all participants who had been diagnosed with HIV/AIDS. The collected information included socio-demographic information, possible route of HIV transmission, HIV-related behaviors, and disease-related characteristics. The socio-demographic information included sex, age, occupation, current marital status, ethnicity, and education level. The possible route of HIV was obtained by an epidemiologic investigation conducted by medical workers, including heterosexual contacts, intravenous drug use, both heterosexual contact and intravenous drug use, and mother-to-child transmission. HIV-related behaviors included needle sharing and the number of casual sexual partners in their lifetime. Disease-related characteristics included the stage of the disease and the history of other sexually transmitted diseases.

### Laboratory Tests

Each participant was confirmed to be infected with HIV by Western blot, according to the instructions of the manufacturer. Each participant was asked to provide 5 ml of venous blood to detect the recent or long-term infection. The plasma samples were isolated from each participant and preserved in a −80°C freezer until analysis.

### LAg EIA

In this study, the HIV status of each participant was assessed by the Kinghawk HIV-1 Lag-Avidity EIA (LAg EIA) (Beijing Kinghawk Pharmaceutical Co., Ltd., Beijing, China) to distinguish between the recent and long-term infections. LAg EIA was performed according to the kit instructions and the National Guideline for Detection of HIV/AIDS (2015 edition). Normalized OD (ODn) values were evaluated for each specimen, using a CAL specimen tested on the same plate as follows: ODn = specimen OD/median CAL OD. The recent or long-term HIV-1 status was evaluated by the ODn value. If ODn ≤ 1.5, the specimen is regarded as a recent infection; otherwise, the specimen is regarded as a long-term infection (>130 days) (18).

### Nucleic Acid Extraction, Amplification, and Sequencing

Total viral nucleic acid was extracted from the 200 μl plasma of each participant using an automatic extraction machine (MagNA Pure LC 2.0 system, Roche, Branchburg, NJ, United States). The reverse transcription-PCR (RT-PCR) was performed to amplify the full-length protease gene in *Pol* region and the first 300 codons of the reverse transcriptase gene, which was described previously (19). The PCR-amplified products were purified from 1% agarose gel and sequenced by Beijing Genomics Research Center Ltd., in China.

The HIV-1 *pol* sequences obtained in the study, together with reference sequences of different subtypes and CRFs, were edited and aligned using ChromasProl.33. The sequence alignments were manually performed by the BIOEDIT Sequence Alignment Editor software (Ibis Biosciences, Carlsbad, CA, United States). HIV-1 genotype subtypes were identified by phylogenetic analysis and determined by FastTree 2.1.8 (http://meta.microbesonline.org/fasttree/) and FigTree v1.4.2 (http://tree.bio.ed.ac.uk/software/figtree/) software packages. The amplification and sequencing analyses were described earlier in detail (19, 20).

### Genotypic Resistance Analysis

Drug resistance mutations were analyzed using the Stanford University HIV Drug Resistance Database (http://hivdb.stanford.edu/). The degree of low or higher drug resistance of one or more drugs was defined as drug resistance (21). All experimental protocols were in accordance with the instructions of the manufacturer.

### Genetic Transmission Network of HIV-1

Genetic distance-based methods were used to analyze the genetic transmission networks. These were performed by comparing sequences, constructing the phylogenetic tree, calculating pairwise distance, and visualizing the network. The detailed process of the genetic transmission network analyses was described earlier (20).

### Statistical Analysis

Categorical variables were compared using the chi-squared (χ^2^) test. Using the presence of TDR as the dependent variable, univariate logistic regression models were used to examine the significance of the association between each covariate and outcome variable. Crude odds ratio (OR) and a 95%CI for socio-demographic information, the possible HIV transmission route, HIV-related risk behaviors, disease-related characteristics, and recent infections by LAg EIA and subtypes of HIV were obtained in the univariate logistic regression models. Due to the sample size of individuals with some of the outcome variables <5, we used Poisson's regressions to estimate the association between any variable having a significance in the respective univariate test (*P* < 0.01) and an outcome variable. Adjusted ORs and their respective 95% CIs of the variables were obtained. All data were analyzed by R 3.6.2. Statistical significance was declared if a two-sided *p*-value was <0.05.

## Results

### Prevalence and Determinants of TDR

A total of 1,595 newly diagnosed PLWH were enrolled in this study, with a successful sequence acquisition in 1,318 (82.63%) cases, including 659 (50%) male and 659 (50%) female. The median age was 29 years, ranging from 1 to 72 years. Most of the participants were peasant (81.41%), of Yi ethnicity (99.01%), married (55.69%), and illiterate (76.86%). The routes of transmission were mostly heterosexual contact, followed by intravenous drug use (18.74%) and mother-to-child transmission (16.62%). A total of 283 (21.47%) cases reported a history of sharing needles, 534 (40.52%) reported a history of casual sexual partners, and 20 (1.52%) reported a history of sexually transmitted diseases other than HIV (Table 1).

**Table 1:** Prevalence and determinants of transmitted drug resistance (TDR) among the participants.

**Variables**	**TDR**	**Rate (%)**	**Total number, *N* = 1,318**	**Crude OR**	**Adjusted OR**
**Sex**					
Female	53	8.04	659	Reference	
Male	54	8.19	659	1.02 (0.69, 1.52)	–
**Age**					
≤ 15	22	9.69	227	Reference	
15~40	68	7.47	910	0.75 (0.45, 1.25)	
>40	16	9.14	175	0.94 (0.48, 1.84)	–
Unknown	1	16.67	6		
**Occupation**					
Peasant	83	7.74	1,073	Reference	
Employed	1	5.88	17	0.75 (0.10, 5.91)	
Students and children	23	10.09	228	1.34 (0.82, 2.18)	–
**Current marital status**					
Married	54	7.36	734	Reference	
Single	37	9.14	405	1.27 (0.82, 1.96)	
Widowed or divorced	6	8.00	75	1.10 (0.46, 2.64)	–
Unknown	10	9.62	104		
**Ethnicity**					
Yi	105	8.05	1,305	Reference	
Han and others	2	15.38	13	2.08 (0.46, 9.50)	–
**Education level**					
Illiteracy	85	8.39	1,013	Reference	
Primary school or above	22	7.21	305	0.85 (0.52, 1.38)	–
**HIV transmission route**					
Heterosexual contact	68	8.27	822	Reference	
Intravenous drug use	16	6.48	247	0.77 (0.44, 1.35)	
Heterosexual contact and intravenous drug use	2	6.67	30	0.79 (0.19, 3.40)	
Mother-to-child transmission	21	9.59	219	1.18 (0.70, 1.97)	–
**Stage of disease**					
HIV	84	6.99	1,201	Reference	Reference
AIDS	23	19.66	117	3.25 (1.96, 5.40)^***^	2.32 (1.44, 3.75)^***^
**History of needle sharing**					
No history of using illicit drugs	72	6.96	1,035	Reference	Reference
Had shared needle for 1~4 times	22	8.91	247	1.31 (0.79, 2.15)	1.12 (0.68, 1.86)
Had shared needle ≥5 times	13	36.11	36	7.56 (3.68, 15.55)^***^	3.89 (2.08, 7.31)^***^
**Number of casual sexual partners in a lifetime**					
0	72	9.18	784	Reference	Reference
1~4	31	7.01	442	0.75 (0.48, 1.16)	0.89 (0.57, 1.40)
≥5	4	4.35	92	0.45 (0.16, 1.13)	0.53 (0.19,1.47)
**History of sexually transmitted diseases except for HIV**					
Never	106	8.17	1,298	Reference	
Ever	1	5.00	20	0.59 (0.08, 4.47)	–
**Recent infections by LAg EIA**					
Yes	3	8.33	36	Reference	
No	104	8.11	1,282	0.97 (0.29, 3.22)	–
**Within genetic transmission network**					
Yes	40	6.93	577	Reference	Reference
No	67	9.04	741	1.34 (0.89, 2.01)	1.27 (0.86, 1.88)
**Subtypes**					
CRF07_BC	100	8.13	1,230	Reference	
CRF08_BC	6	10.91	55	1.38 (0.58, 3.31)	
CRF77_cpx	1	3.70	27	0.44 (0.06, 3.24)	–
Others	0	0.00	6		

*** *P < 0.001*.

The prevalence of TDR among newly diagnosed PLWH was 8.12% (107/1,318). In the multivariate logistic regression model, we found that participants who were in the stage of AIDS (adjusted OR: 2.32, 95%CI: 1.44–3.75) and had a history of sharing a needle ≥5 times (adjusted OR: 3.89; 95%CI: 2.08–7.31; reference group: no history of illicit drugs) were more likely to have an increased risk of TDR among newly diagnosed PLWH (*P* < 0.05) (Table 1).

### Recent Infections and Distribution of HIV-1 Genotypes

Of the 1,318 newly diagnosed PLWH, 36 (2.73%) were considered as having recently been infected (within 130 days) by LAg EIA. Of the 1,318 amplified samples, circulating recombinant form (CRF) 07_BC (93.32%, 1,230/1,318) was the predominant genotype, followed by CRF08_BC (4.17%, 55/1,318) and CRF77_cpx (2.05%, 27/1,318) (Supplementary Figure 3).

### Prevalence and Resistance Level of TDR-Associated Mutations

**Figure 1:**
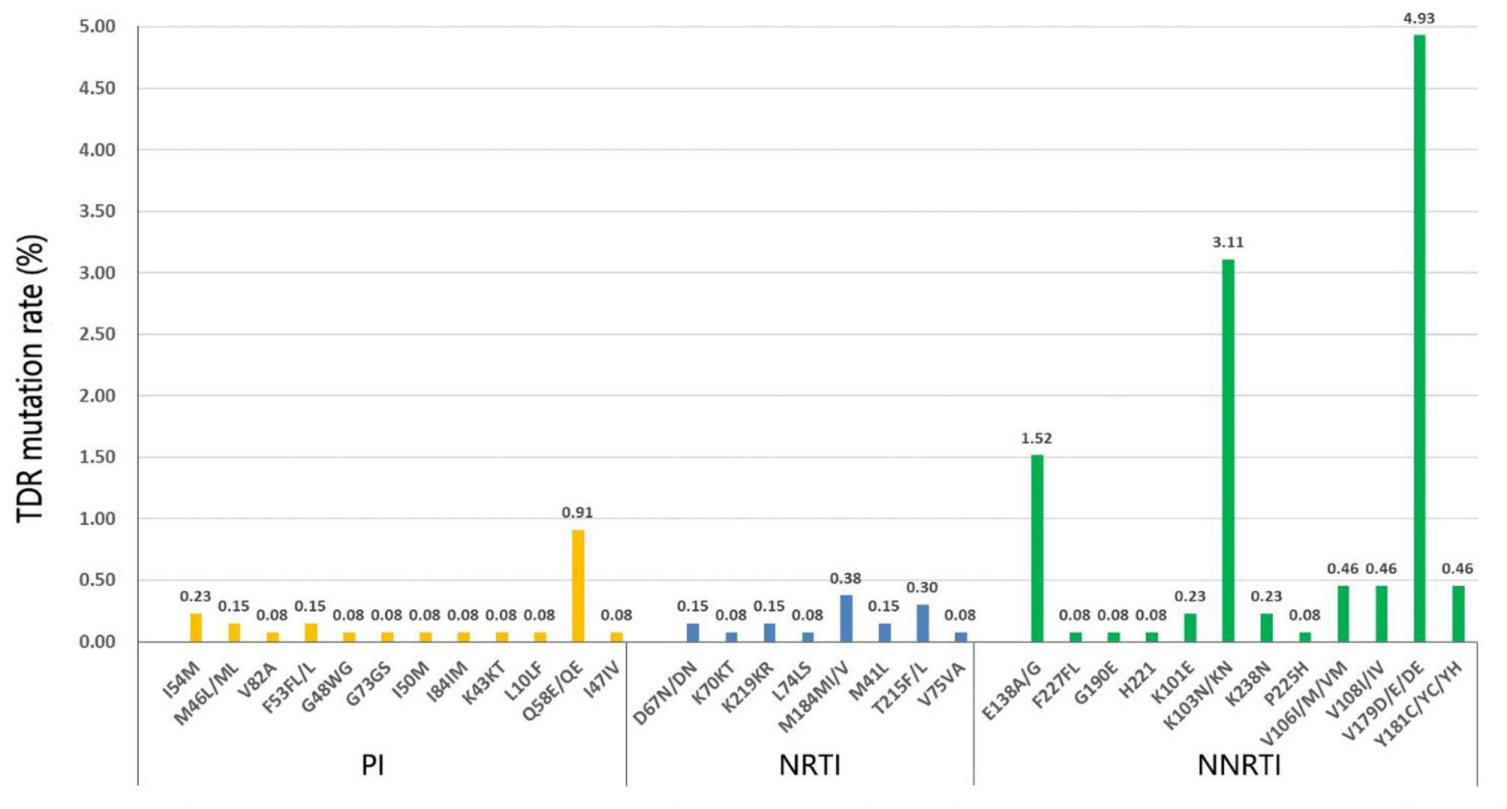
Different HIV-1 transmitted drug resistance (TDR) mutations to PIs, NRTIs, and NNRTIs.

The prevalence of TDR for non-nucleoside reverse transcriptase inhibitors (NNRTIs), NRTIs, and PIs mutation patterns was 6.30% (83/1,318), 0.91% (12/1,318), and 1.67% (22/1,318), respectively (Figure 1). There were 154 HIV-1 TDR-associated mutations to NNRTIs, 18 to NRTIs, and 27 to PIs. The most frequent NNRTI-associated mutation in our study was V179D/E/DE (4.93%), followed by K103N/KN (3.11%) and E138A/G (1.52%). M184MI/V (0.38%) was the most prevalent NRTI-associated mutation, followed by T215F/L (0.30%). Q58E/QE (0.91%) was the major prevalent PI-associated mutation, followed by I54M (0.23%). The majority of HIV-1 strains with TDR mutations exhibited the degree of potential low-level resistance to NNRTI, ascribed to V179D/E/DE.

The HIV-1 resistance level to 20 commonly used antiretroviral drugs was analyzed (Supplementary Table 1). As for the NNRTI resistance, the majority of the HIV-1 strains showed high-level resistance to NVP (3.95%) and EFV (3.64%), and the main mutation was K103N/KN (data not shown). Most exhibited a potential low-level resistance to ETR (6.45%), EFV (5.84%), NVP (5.39%), and RPV (4.86%), and the main mutation was G190A. As for the PI resistance, HIV-1 strains exhibited more TDR frequencies to NFV, and most showed a low-level resistance to TPV/r (1.06%). As for the NRTI resistance, the HIV-1 strains only exhibited high-level resistance to FTC (0.38%) and 3TC (0.38%), while they show a low and potential low-level resistance to AZT, D4T, and DDI.

### Analysis of HIV-1 Genetic Transmission Network

**Figure 2:**
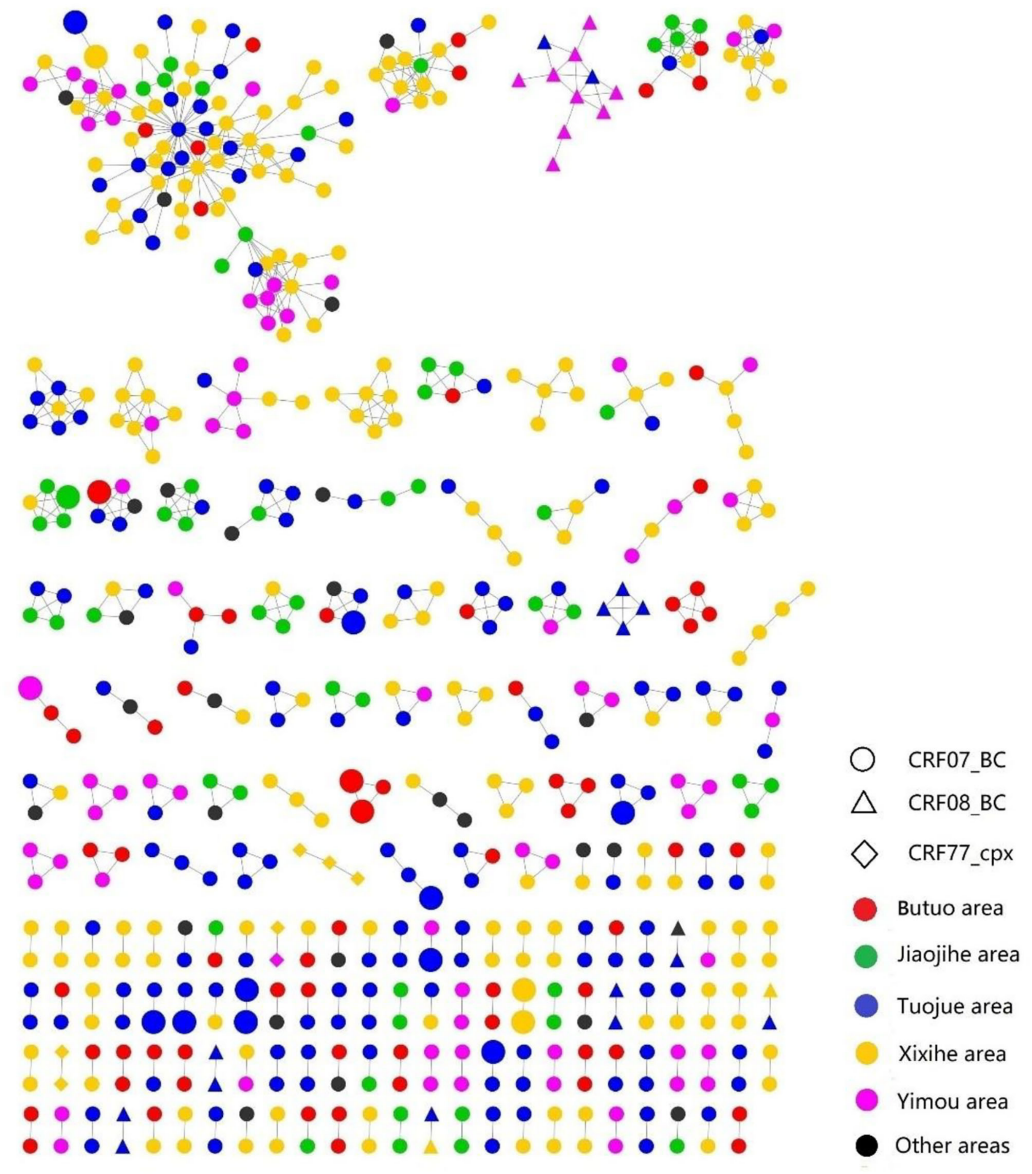
The transmission networks of HIV-1 newly diagnosed PLWH by HIV-1 subtypes and locations; the large shapes represented recent infections within 130 days by LAg EIA.

**Figure 3:**
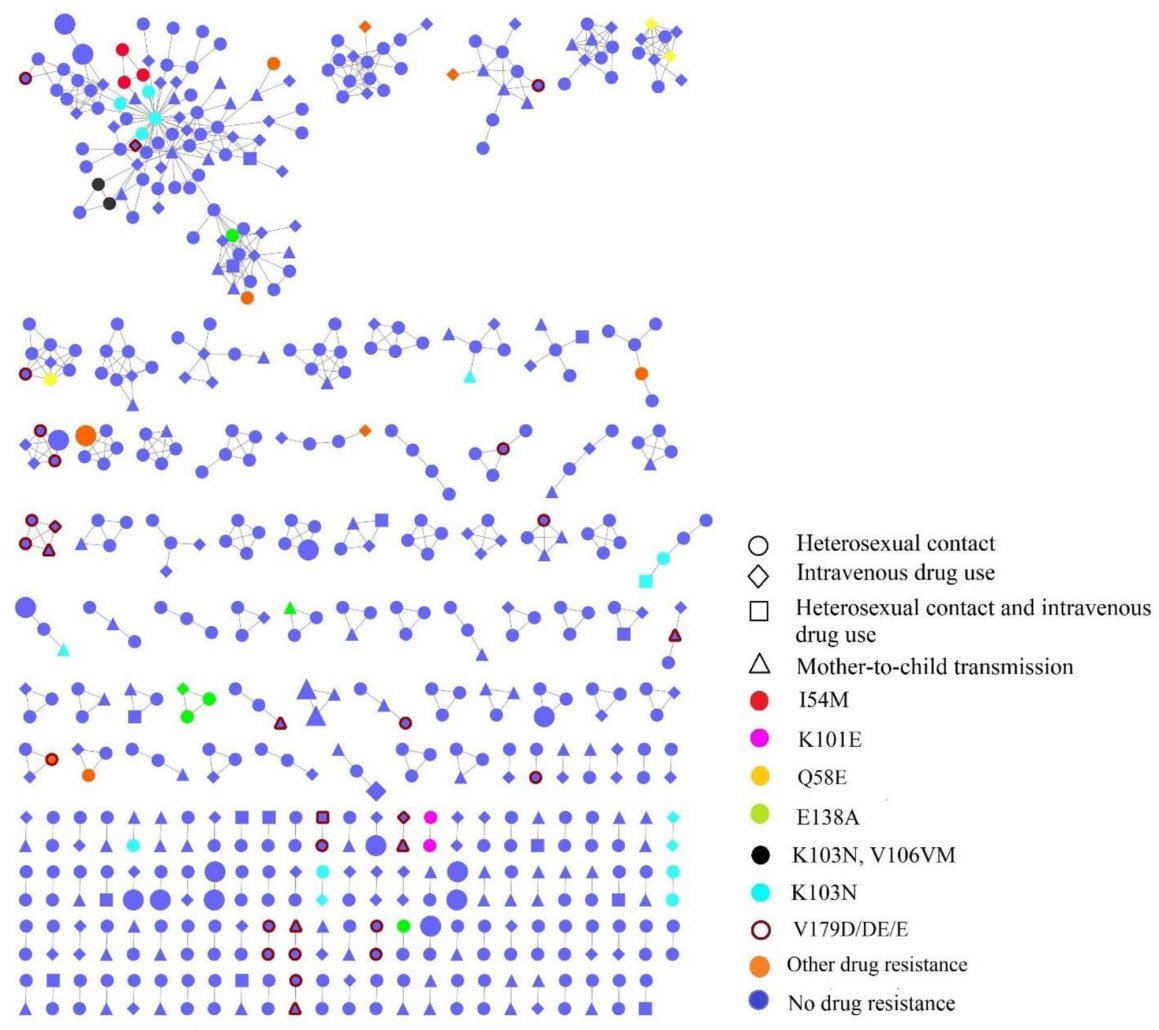
The transmission networks of HIV-1 newly diagnosed people living with HIV (PLWH) by HIV transmission routes and TDR mutations; the large shapes represented recent infections within 130 days by LAg EIA.

A total of 577 (43.78%, 577/1,318) *pol* sequences were identified and used for the genetic transmission network analysis. A total of 171 clusters were observed at a genetic distance of 0.013, resulting in 171 clusters (Figures 2, 3). Among these clusters, 212 sequences (36.7%) were only linked to one *pol* sequence, and 365 (63.3%) were linked to more than two *pol* sequences. The biggest cluster involved 91 *pol* sequences. Of the sequences in the network, 543 (44.15%, 543/1,230) were CRF07_BC *pol* sequences, 27 (49.09%, 27/55) were CRF08_BC, and 7 (25.93%, 7/27) were CRF77_cpx (Figure 2).

Individuals in the networks were more likely to come from the same village or town. Of 36 recently infected individuals, 18 (50%, 18/36) were in the networks, among which eight (44.44%, 8/18) were genetically linked to another recently infected individual. As for the HIV transmission routes, heterosexual contact and intravenous drug use were often cross-linked (Figure 3).

Individuals with the risk behaviors of both heterosexual contact and intravenous drug use (53.33%, 16/30) were more likely to be in the network. We observed that individuals with TDR-associated mutations were more likely linked to those with the same mutations. Besides, 37.38% (40/107) of individuals carrying TDR were involved in the network, and K103N (42.5%, 17/40) was the most frequent TDR-associated mutation, followed by E138A (15%, 6/40), I54M (7.5%, 3/40), and Q58E (7.5%, 3/40). Twenty-nine individuals in the networks had potential low-level resistant mutations (V179D/DE/E).

## Discussion

When HIV replicates under a non-adapted or not well-taken ART, HIV drug resistance emerges. TDR of HIV strains is associated with a suboptimal virologic response to initial ART, and the TDR test is beneficial to guide the regimen selection to optimize the treatment combination and improve the efficiency of ART in a population with a high mortality rate (22). The prevalence of TDR among PLWH in Liangshan was 8.12%, which was higher than other epidemic areas in China (3.7–6.12%) (23–25) and higher than six sub-Sahara African countries with a similar period of ART roll-out as China (26). Since the stage of AIDS and history of frequent needle sharing were risk factors for TDR, strengthening the monitor of TDR among AIDS patients and HIV-infected drug users in Liangshan is perhaps important than ever before.

The TDR to NRTIs was not common in our study. The results are similar to the findings of other studies (27, 28). The main reason might be the use of a TDF- or AZT-based regimen in place of the D4T-based regimen that is associated with a higher adverse drug reaction and drug resistance (29). In the past few years, China's National Free Antiretroviral Treatment Program (NFATP) expanded the use of TDF according to the WHO guideline and gradually phased out D4T in favor of TDF for the first-line ART (30). TDF and initiated TDF-based regimens are associated with lower drug resistance and lower TDR, as newer ART agents are more effective and safer than older nucleoside reverse transcriptase inhibitor agents. The prevalence of TDR to current first- and second-line regimens was 0.91–6.30% and 1.67%, indicating a high sensitivity to the first-line regimen among most newly diagnosed PLWH. Therefore, even if the drug-resistant test cannot become a routine test, there is no need to expand the second-line treatment for PLWH. These findings are of great significance for both the clinical practice of HIV treatment and public health policy-making.

Our study found that the two NRTI mutations (M184MI/V and T215F/L) had a higher prevalence than other TDR mutations in this population. Since 2016, all newly diagnosed PLWH are required to receive ART in China, and the massive scale-up of ART that could not ensure easy access to well-tolerated antiretroviral drugs to any PLWHA has inevitably increased the threat of TDR (28). One point of concern is the free NRTI drug 3TC, which had been listed and widely used in the first- and second-line ART regimens for the past 20 years in China. It is reported that the M184MI/V mutation showed a high- and intermediate-level resistance to 3TC that reduces the replicative capacity of reverse transcriptase (31, 32). Although it is still debatable whether continued use of 3TC provides actual benefit, it is still lacking a fully active multitherapy in the presence of the M184V mutation, which would lead to a greater risk for virologic failure and may be related to higher TDR in newly diagnosed PLWH (33). Previous studies in China also observed a high percentage of M184 MI/V in ART-naive individuals across the country, indicating the importance of modifying the regimen accordingly (28). The resistance mutations to 3TC should be closely monitored to prevent the threat of TDR.

In the genetic transmission network analysis, some certain demographic and transmission characteristics are worthy of attention. First, a large number of transmission clusters were identified in the network, with 43.78% of the analyzed samples. This is not uncommon in HIV epidemics as a smaller population is often part of wide transmission networks (25, 34), suggesting that many individuals were infected with HIV in this area. Large clusters mostly comprising more than two sequences were dominant over small clusters, which is consistent with heterosexual contact being the main route of transmission in newly diagnosed PLWH. Second, the majority of PLWH in the clusters were from different villages or towns. Unlike other minority groups in China, the Yi minority in Liangshan still maintains cultural traditions and values that condone casual sex (3, 35, 36). Casual sex among people of the Yi minority usually occurs during social events/gatherings (wedding, marketing date, Yi festivals, etc.), which would not limit their sexual partners to their place of residence (35, 36). Third, 50% (18/36) of the recently infected individuals who were involved in the network were infected through heterosexual contact. Further effective and tailor-made programs are needed to reduce sexual transmission among sexually active individuals.

In terms of TDR-associated mutations in transmission clusters, 37.38% of individuals carrying TDR were involved in the network. Specifically, 16 transmission clusters that contained individuals carrying TDR had a transmission relationship with each other; the remaining 17 clusters, involving individuals carrying TDR in the network, were independent, suggesting the serious spread of TDR-associated mutations. K103N (42.5%, 17/40) were the most frequent TDR-associated mutations and were in five clusters in which they linked with those carrying the same mutations. K103N mutations might have been in circulation for a while and were unsuspectingly propagated onward in the community as new infections. This finding was also observed in other undeveloped (37) and developed settings (38, 39). Routine TDR testing is needed to reduce TDR-associated mutations.

The proportion of recent infections (within 130 days) among all newly diagnosed PLWH was only 2.73% in this area, which also deserves concern. The proportion of recent infections was lower than that in other areas with a high rate of HIV in China (40, 41) and those in other countries (40, 42), indicating that most of the newly diagnosed PLWH were not detected in time. HIV might be spread by many individuals unaware of their infection, leading to a widespread of HIV in this area. Early detection of HIV should be advocated in this area, so as to effectively control the spread and prevalence of HIV.

Several limitations should be highlighted in this study. First, this study was a cross-sectional survey; time-based sequences and cause-effect relationships among these variables cannot be established. Second, patients in this study were newly diagnosed, but we cannot confirm the time of initial infection, so it is hard to determine the time sequence in the genetic transmission networks. Third, to fully understand the determinants of the genetic transmission network, more PLWH in this area should be enrolled, rather than newly diagnosed PLWH. Fourth, our study was conducted in areas with a high HIV prevalence, so caution should be taken when generalizing the findings to other areas or contexts. Despite the aforementioned limitations, the main strength of this study is the use of samples collected from the HIV screening samples, which represents the area with a high rate of HIV in China.

## Conclusion

To our knowledge, this is the first effort to investigate the prevalence and molecular epidemiology of TDR on the basis of whole-population screening. Our data suggest that a concentrated epidemic is characterized by a predominant HIV-1 CRF07_BC, a relatively high level of TDR, and many transmission clusters. The high prevalence of long-term HIV-1-infected individuals and a large proportion of individuals within the genetically transmitted networks suggest that HIV-1 may be propagated by HIV-1-infected individuals who were unaware of their HIV-1 status. Therefore, targeted HIV-1 prevention, early identification, and monitoring of resistance are warranted to reduce TDR and prevent HIV-1 transmission in areas with a high rate of HIV.

## Data Availability Statement

The raw data supporting the conclusions of this article will be made available by the authors, without undue reservation.

## Ethics Statement

The study protocol was approved by the Ethics Committee of Sichuan Center for Disease Prevention and Control. Written informed consent to participate in this study was provided by the participants' legal guardian/next of kin.

## Author Contributions

DY, BY, SY, PJ, and SL had taken a principal role in the conception of ideas, writing the protocol, developing methodologies, analyses and write-up of the article, and drafted the manuscript. ZW and PJ contributed to the proposal writing and made a critical revision to the paper and manuscript. PJ, SY, and ML contributed to the write-up of the study protocol and revised the paper. YL, LY, YH, LS, YZ, LA, MC, CZ, LL, and LZ supervised the study and edited the manuscript. ZW revised the paper. All authors read and approved the final manuscript.

## Funding

This research was funded by the National Natural Science Foundation of China (81703279); Sichuan Science and Technology Program (2019YJ0148); National Major Science and Technology Projects of China: Study on the assessment technology and prediction model of AIDS epidemic in China (2017ZX10201101), Science and Technology Project of Sichuan Provincial Health Committee (20PJ121), and the International Institute of Spatial Lifecourse Epidemiology (ISLE). PJ and SY, Co-directors of the ISLE, thank the West China School of Public Health and West China Fourth Hospital in Sichuan University, the Chinese Center for Disease Control and Prevention, the Netherlands Organization for Scientific Research, and the Royal Netherlands Academy of Arts and Sciences for funding the ISLE and supporting ISLE's research activities.

## Conflict of Interest

The authors declare that the research was conducted in the absence of any commercial or financial relationships that could be construed as a potential conflict of interest.

## Publisher's Note

All claims expressed in this article are solely those of the authors and do not necessarily represent those of their affiliated organizations, or those of the publisher, the editors and the reviewers. Any product that may be evaluated in this article, or claim that may be made by its manufacturer, is not guaranteed or endorsed by the publisher.

## Abbreviations

HIV: Human immunodeficiency virus
AIDS: Acquired Immune Deficiency Syndrome
ART: Antiretroviral therapy
PLWH: People living with HIV
TDR: Transmitted drug resistance
LAg EIA: Lag-Avidity EIA
RT-PCR: Reverse transcription-polymerase chain reaction
OR: Odds ratios
CI: confidence interval
NNRTI: nonnucleoside reverse transcriptase inhibitor
NRTI: nucleoside reverse transcriptase inhibitor
PI: protease inhibitor
TDF: Tenofovir
3TC: Lamivudine
NVP: Nevirapine
EFV: Efavirenz
AZT: Zidovudine
LPV/r: Fosamprenavir/ritonavir
ATV/r: Atazanavir/ritonavir
DRv/r: Darunavir/ritonavir
FPV/r: Fosamprenavir/ritonavir
IDV/r: Indinavir/Ritonavir
NFV: Nelfinavir
SQV/r: Ritonavir-boosted saquinavir
TPV/r: Tipranavir/ritonavir
ABC: Abacavir
D4T: Stavudine
DDI: Didanosine
FTC: Emtricitabine
ETR: Etravirine
RPV: Rilpivirine.
