# Supplementary material for: Prevalence and Molecular Epidemiology of Transmitted Drug Resistance and Genetic Transmission Networks Among Newly Diagnosed People Living With HIV/AIDS in a Minority Area, China
Source: Front Public Health. 2021 Oct 11;9:731280. doi: 10.3389/fpubh.2021.731280 (PMC8542729; doi:10.3389/fpubh.2021.731280)
Supplement: Supplementary file 1 [file Data_Sheet_1.docx]

Supplementary Material

## Supplementary Figures


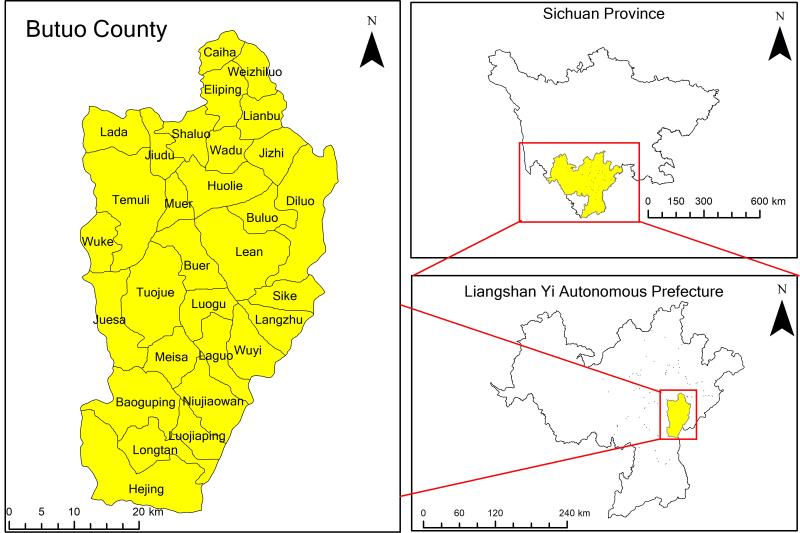


**Figure S1.** Location of Butuo county, China


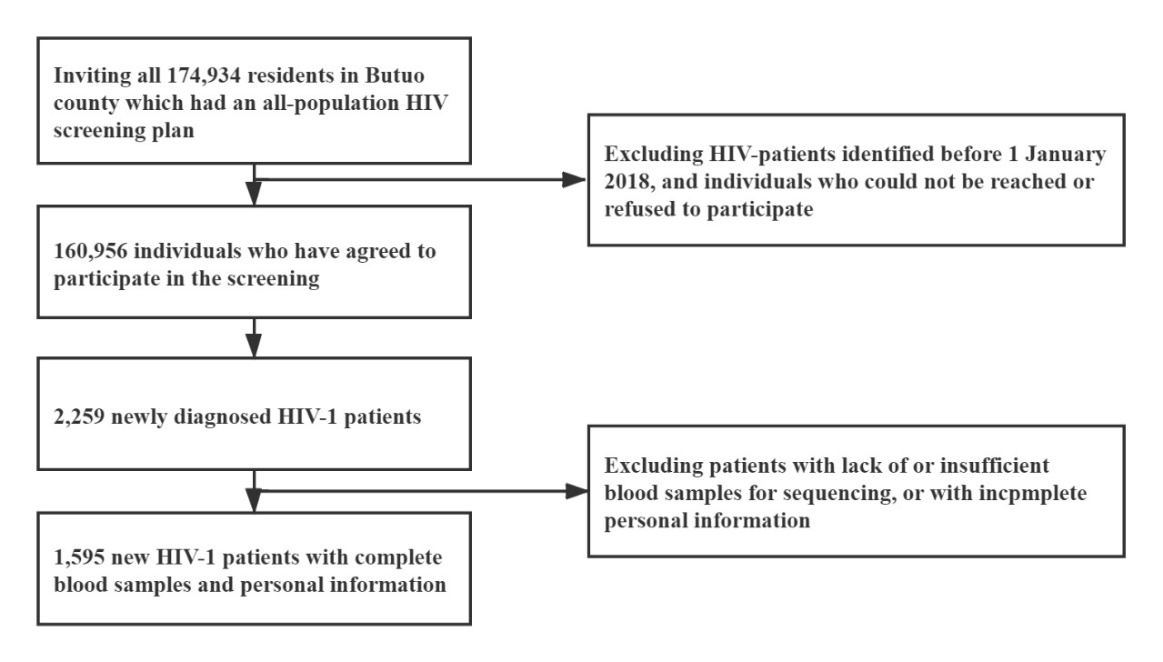


**Figure S2.** Sampling flowchart


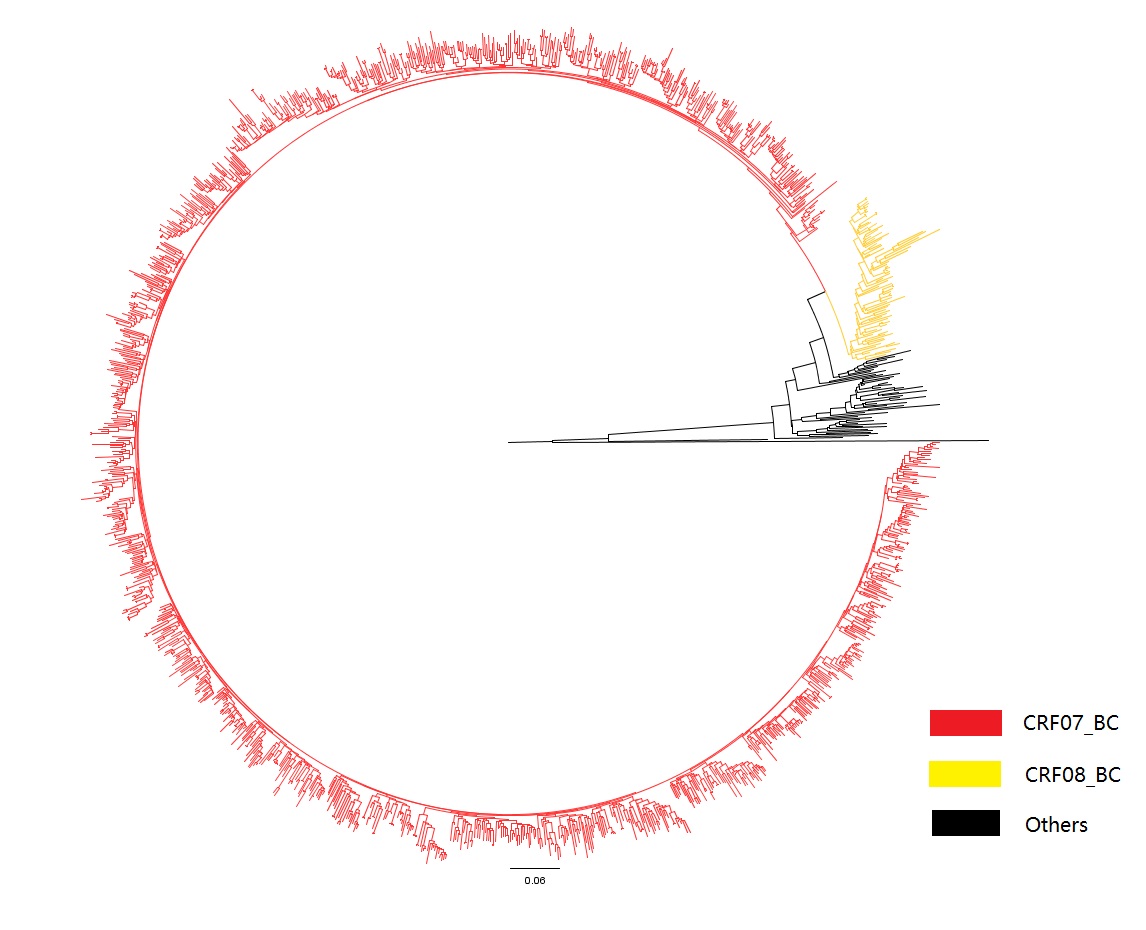


**Figure S3.** Phylogenetic tree analyses of the HIV-1 *pol* sequences of the participants; CRF07_BC accounted for 93.32% of the HIV-1 *pol* sequences; CRF08_BC accounted for 4.17% of the HIV-1 *pol* sequences; others were CRF77_cpx and reference strains

# Supplementary Tables

**Table S1.** Levels of HIV-1 TDR associated mutations to different ART drug among 1,318 newly diagnosed HIV/AIDS patients

|  | High | | Intermediate | | Low | | Potential-low | |
| --- | --- | --- | --- | --- | --- | --- | --- | --- |
| Drugs | Frequency | % | Frequency | % | Frequency | % | Frequency | % |
| **PI** |  |  |  |  |  |  |  |  |
| ATV/r | 0 | 0.00 | 0 | 0.00 | 4 | 0.30 | 6 | 0.46 |
| DRV/r | 0 | 0.00 | 0 | 0.00 | 3 | 0.23 | 1 | 0.08 |
| FPV/r | 3 | 0.23 | 1 | 0.08 | 2 | 0.15 | 3 | 0.23 |
| IDV/r | 0 | 0.00 | 1 | 0.08 | 5 | 0.38 | 3 | 0.23 |
| LPV/r | 0 | 0.00 | 1 | 0.08 | 4 | 0.30 | 2 | 0.15 |
| NFV | 0 | 0.00 | 2 | 0.15 | 7 | 0.53 | 15 | 1.14 |
| SQV/r | 0 | 0.00 | 0 | 0.00 | 7 | 0.53 | 3 | 0.23 |
| TPV/r | 0 | 0.00 | 1 | 0.08 | 14 | 1.06 | 3 | 0.23 |
| **NRTI** |  |  |  |  |  |  |  |  |
| ABC | 0 | 0.00 | 1 | 0.08 | 5 | 0.38 | 0 | 0.00 |
| AZT | 0 | 0.00 | 1 | 0.08 | 6 | 0.46 | 2 | 0.15 |
| D4T | 0 | 0.00 | 3 | 0.23 | 5 | 0.38 | 2 | 0.15 |
| DDI | 0 | 0.00 | 1 | 0.08 | 2 | 0.15 | 8 | 0.61 |
| FTC | 5 | 0.38 | 0 | 0.00 | 0 | 0.00 | 1 | 0.08 |
| 3TC | 5 | 0.38 | 0 | 0.00 | 0 | 0.00 | 1 | 0.08 |
| TDF | 0 | 0.00 | 0 | 0.00 | 2 | 0.15 | 0 | 0.00 |
| **NNRTI** |  |  |  |  |  |  |  |  |
| DOR | 2 | 0.15 | 8 | 0.61 | 7 | 0.53 | 9 | 0.68 |
| EFV | 48 | 3.64 | 6 | 0.46 | 6 | 0.46 | 77 | 5.84 |
| ETR | 1 | 0.08 | 5 | 0.38 | 5 | 0.38 | 85 | 6.45 |
| NVP | 52 | 3.95 | 6 | 0.46 | 9 | 0.68 | 71 | 5.39 |
| RPV | 4 | 0.30 | 6 | 0.46 | 23 | 1.75 | 64 | 4.86 |

TDF: tenofovir; 3TC: lamivudine; NVP: nevirapine; EFV: efavirenz; AZT: Zidovudine; LPV/r: fosamprenavir/ritonavir; ATV/r- atazanavir/ritonavir; DRv/r Darunavir/ritonavir, FPV/r: fosamprenavir/ritonavir; IDV/r: Indinavir/Ritonavir; NFV: nelfinavir; SQV/r: ritonavir-boosted saquinavir; TPV/r: tipranavir/ritonavir; ABC: Abacavir; D4T: Stavudine; DDI: didanosine; FTC: Emtricitabine; ETR: etravirine; RPV: rilpivirine. PI: protease inhibitor; NRTI: nucleoside reverse transcriptase inhibitor; NNRTI: nonnucleoside reverse transcriptase inhibitor
